# Core issues, case studies, and the need for expanded Legacy African American genomics

**DOI:** 10.3389/fgene.2023.843209

**Published:** 2023-06-08

**Authors:** Fatimah Jackson, Carter Clinton, Jennifer Caldwell

**Affiliations:** ^1^ Department of Biology, Howard University, Washington, DC, United States; ^2^ Department of Biology, North Carolina State University, Raleigh, NC, United States; ^3^ Pennington Biomedical Research Center, Louisiana State University, Baton Rouge, LA, United States

**Keywords:** bioethics, NYABG, Gullah Geechee, genomic equity, genetic databases

## Abstract

**Introduction:** Genomic studies of Legacy African Americans have a tangled and convoluted history in western science. In this review paper, core issues affecting African American genomic studies are addressed and two case studies, the New York African Burial Ground and the Gullah Geechee peoples, are presented to highlight the current status of genomic research among Africa Americans.

**Methods:** To investigate our target population’s core issues, a metadatabase derived from 22 publicly accessible databases were reviewed, evaluated, and synthesized to identify the core bioethical issues prevalent during the centuries of the African American presence in North America. The sequence of metadatabase development included 5 steps: identification of information, record screening and retention of topic relevant information, identification of eligibility via synthesis for concept identifications, and inclusion of studies used for conceptual summaries and studies used for genetic and genomic summaries. To these data we added our emic perspectives and specific insights from our case studies.

**Results:** Overall, there is a paucity of existing research on underrepresent African American genomic diversity. In every category of genomic testing (i.e., diagnostic, clinical predictive, pharmacogenomic, direct-to-consumer, and tumor testing), African Americans are disproportionately underrepresented compared to European Americans. The first of our case studies is from the New York African Burial Ground Project where genomic studies of grave soil derived aDNA yields insights into the causes of death of 17^th^ and 18^th^ Century African Americans. In the second of our case studies, research among the Gullah Geechee people of the Carolina Lowcountry reveals a connection between genomic studies and health disparities.

**Discussion:** African Americans have historically borne the brunt of the earliest biomedical studies used to generate and refine primitive concepts in genetics. As exploited victims these investigations, African American men, women, and children were subjected to an ethics-free western science. Now that bioethical safeguards have been added, underrepresented and marginalized people who were once the convenient targets of western science, are now excluded from its health-related benefits. Recommendations to enhance the inclusion of African Americans in global genomic databases and clinical trials should include the following: emphasis on the connection of inclusion to advances in precision medicine, emphasis on the relevance of inclusion to fundamental questions in human evolutionary biology, emphasis on the historical relevance of inclusion for Legacy African Americans, emphasis on the ability of inclusion to foster expanded scientific expertise in the target population, ethical engagement with their descendants, and increase the number of science researchers from these communities.

## Introduction: status of genomic studies among peoples of African descent

The representation and treatment of African Americans in the biomedical, anthropological, and genomic literature has a tumultuous history. This systematic review discusses the importance of increasing genomic research performed on and for populations from underrepresented ancestries and the significance this enrichment would bring to our global genomic databases and accelerate the progress of our science. If all populations were fairly represented, the potential benefits of genomic research (e.g., better understanding of disease etiology, earlier detection ad diagnostics, rational drug design, and increased clinical care) would be available to underrepresented groups such as peoples of African descent (Fatumo et al., 2022) ***.*** However, the current persistent Eurocentric bias in genomics and genetics extends through all levels of the discipline, including affecting the utility of polygenic risk scores in disease studies (Martin et al., 2019) ***,*** which still have limitations for peoples of African descent since such studies are rooted in GWAS databases with a North Atlantic ancestry-centered ascertainment bias (Gultig, 2023) ***.***

In this review, we focus on Legacy African American populations, i.e., indigenous African Americans living in the United States, as a subset of peoples of recent African descent, and the reoccurring deficit of meaningful and adequate genomic studies available on this group. Legacy African Americans are the historic African American descendants of the heritage of American Slavery, Jim Crow segregation, and institutionalized racial discrimination. African American genomics is best framed within the context of continental African genomic diversity. In this review, we explore the current core issues in African American genomic studies, using two specific case studies. Here we show that the addition of genomic data from the remains of deceased individuals is a valuable and necessary adjunct to those data derived from the biological samples of living individuals and that the aDNA provides more insights than simply relying on skeletal and dental assessments alone. Using the historic New York African Burial Ground (NYABG) and the contemporary Gullah Geechee peoples of the Coastal Sea Islands and South Carolina and Georgia Lowcountry, we demonstrate the potential benefits of collecting and exploring ancient DNA (aDNA) and modern DNA samples to create a robust database capable of stimulating future research on Legacy African Americans and beginning to bring parity to genomic inquiries.

Who are Legacy African Americans? According to historical sources (Eltis and Richardson, 2015; Eltis et al., 2017) ***,*** their deepest ancestral origins go back to diverse regions of continental Africa. From 1,501 to 1867, enslaved Africans were forcibly and brutally embarked mainly from eight coastal regions of Africa. According to these historical records, 5.7% embarked from Senegambia, 3.2% from Sierra Leone, 2.7% from the Windward Coast, 9.6% from the Gold Coast, 16.1% from the Bight of Benin, 12.3% from the Bight of Biafra, 46.3% from West Central Africa, and 4.1% from Southeast Africa. Prior to the aggregation of kidnapped Africans at these export sites, the Africans were part of local empires and kingdoms throughout the continent. However, the West Central African coast was the largest slaving and embarkation region throughout most of the trans-Atlantic trade in enslaved Africans (Fortes-Lima and Verdu, 2021) ***,*** and it focused mainly on groups living south of the Congo River. The Gold Coast, the Bight of Benin, and the Bight of Biafra became increasingly prominent slave collecting and embarkation regions after the mid-17th century, as the trans-Atlantic trade in enslaved Africans expanded with the growth of the Plantation Economy in the Americas.

In two recent articles (Caldwell and Jackson, 2021; Jackson, 2021), Legacy African Americans are identified as the current 40+ million Black Americans with multigenerational backgrounds (legacies) of extensive contact with the North American social, cultural, economic, and legal environments. During these 400 years of exposure or approximately 16 generations (25 years per generation) of direct contact with American Slavery, Jim Crow racism and segregation, disparate health and educational opportunities, this background has uniquely shaped both their genomes and epigenomes (Jackson et al., 2018a; Jackson et al., 2018b) ***.*** African Americans are the third largest ethnic group in the United States and are the results of various admixture events, with today showing common ancestry with Africans (∼82.1%), Europeans (∼16.7%), and Native Americans (∼1.2%) (Baharian et al., 2016)**.**

Recent 20th century migrations, like the 1st and 2nd Great Migrations of African Americans, initiated greater intra-group genetic homogeneity despite populations being initially geospatially distant. For example, Detroit, MI attracted African American migrants from Louisiana and the Mississippi Delta. Chicago, IL disproportionately attracted Legacy African Americans from five counties in Mississippi. Los Angeles, CA attracted African Americans from east Texas and Louisiana with some stopping to found previously predominantly African American towns such Dearfield, CO, Nicodemus, KA, and McNary, AZ. Predominantly African American town are part of the history of America. Today, only thirteen historical African American towns survived, but their legacy of economic and political freedom is well remembered. The Oklahoma towns of Boley, Brooksville, Clearview, Grayson, Langston, Lima, Red Bird, Rentiesville, Summit, Taft, Tatums, Tullahassee, and Vernon, for example, attest to the historic settlement patterns of Legacy African Americans. African American-founded towns remained predominantly African American demographically until the towns were disbanded. Increasing urbanization of Legacy African Americans facilitated gene flow between microethnic groups that had been previously distinct local communities with their own unique biological histories, subsistence patterns, and distinct African cultural retentions from the period of enslavement and its aftermath (*see* (Berlin, 2010)) ***.***

## Methods and materials

As a systematic review, we accessed a wide variety of databases in this study to develop a megadatabase. The components of this mega database include.

• TransAtlantic Slave Voyages Database. (https://www.slavevoyages.org/voyage/database) (Slave Voyages Database).
• Accessible Archives Database https://www.accessible.com/accessible/preLog (Accessible Archives)
• AnthroSource, (https://anthrosource.onlinelibrary.wiley.com) (AnthroSource Database),
• Applied Social Sciences Index and Abstracts (ASSIA), (https://about.proquest.com/en/products-services/ASSIA-Applied-Social-Sciences-Index-and-Abstracts/) (Sociological Abstracts Database),
• socINDEX,https://proxy.library.emory.edu/login?url=http://search.ebscohost.com/login.aspx?authtype=ip,uid&profile=ehost&defaultdb=sih) (Emory university)
• Social Sciences Full Text (Wilson Web), (https://www.library.nd.edu/database/4qBOLHGnSwwM0ekI4SUkKC) (Hesburgh libraries),
• Google Scholar, (https://scholar.google.com/) (Google Scholar Search Engine),
• GenBank, (https://www.ncbi.nlm.nih.gov/genbank/) (GenBank),
• 1000 Genomes Project, (https://en.wikipedia.org/wiki/1000_Genomes_Project) (1000 Genomes Project),
• Online Mendelian Inheritance in Man (OMIM), (https://www.omim.org/) (OMIM),
• NCIB Reference Sequence (RefSeq) (https://www.ncbi.nlm.nih.gov/refseq/) (NCIB Reference Sequence)
• The World Bank Database (WHO), https://databank.worldbank.org/databases/africa) (The World Bank Database)
• Open Data for Africa, (https://dataportal.opendataforafrica.org/) (Open Data for Africa),
• African American Biographical Database, (https://aabd.chadwyck.com/) (The African American Biographical Database),
• African American Home Movie Archive, (https://www.aahma.org/) (African American Home Movie Archive),
• African American Odyssey, (https://memory.loc.gov/ammem/aaohtml/aohome.html) (Hine et al., 2010),
• Afro-American Genealogical Research: Introduction (https://guides.loc.gov/african-american-genealogical-research) (Afro-American Genealogical Research, 2020)
• Records of the Continental and Confederation Congresses and the Constitutional Convention (https://www.archives.gov/research/guide-fed-records/groups/360.html) (National Archivesa)
• Annals of Congress, Vol. 1: 1st through 18th (https://memory.loc.gov/ammem/amlaw/lwaclink.html) (The Library of Congress)
• RG 233: Records of the United States House of Representatives (https://www.archives.gov/research/guide-fed-records/groups/233.html) (National Archivesb)
• American Freedmen’s Inquiry Commission, (http://www.shfg.org/resources/Documents/7-Strickland.pdf) (American Freedmen’s Inquiry Commission), and
• Sociological Abstracts, (https://proquest.libguides.com/socabs) (Sociological Abstracts)**.**

**FIGURE 1:**
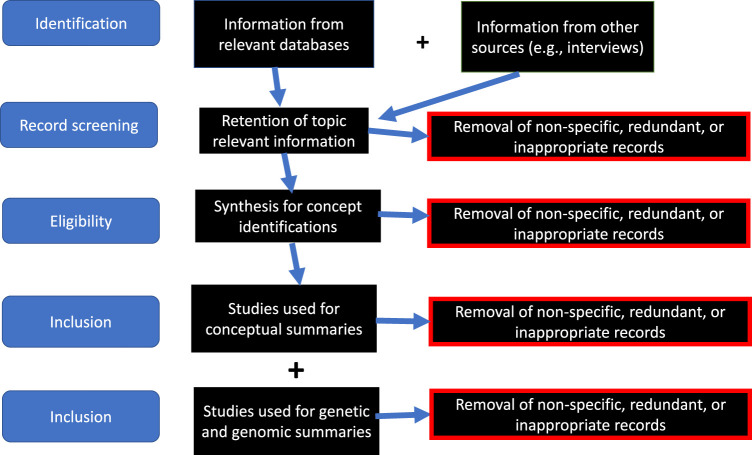
Sequence of development of metadatabase used in this study. All datatbases were publicly available; our strategy identified the most relevant information, refined and curated it, subjected it to uniformity procedures, and excluded al irrelevant data.

Data from these databases were explored and integrated into our discussion of an overall set of concepts and two specific case studies of the bioethics of African American genetics. We synthesized data from these sources as well as our emic perspectives to identify the core issues prevalent during the centuries of the African American presence in North America and germane to the bioethics of genomic biomedical research in this population. The sequence of our uses of these online databases are depicted in Figure 1. Our coordinated review of the constituent databases and other sources of relevance included five steps: identification of information, screening and data-transformation of the records, determination of underlying African American conceptual issues on genetics and genomics, and incorporation of existing genetic and genomic data on Legacy African Americans. Excluded from our consideration were studies on non-Legacy African Americans, studies that did not consider continental Africans, and studies that did not test specific genetic or genomic hypotheses.

## Case studies in African American genomics

### Case #1: New York African Burial Ground (NYABG)

The NYABG is the country’s oldest and largest burial site of free and enslaved Africans ever discovered. Its origin dates to ∼1,640, with a closing date around 1797. The site spans 6.6 acres across the New Amsterdam Colony or present-day New York City (NYC) (GSA)**.** The initial use of the burial ground coincides with the establishment of the Negro Frontier, a free African community just outside of the New Amsterdam Colony. A community that needed a place nearby to bury their loved ones without having to carry them beyond the Colony’s boundary walls or paying a fee. The NYABG was rediscovered in 1991, when 419 skeletons were unearthed during the construction of a federal building at 290 Broadwayin Lower Manhattan. Researchers at Howard University performed robust analyses and generated initial reports on the skeletal biology, history, and archaeology of the site and the population. At the conclusion of this landmark project, the skeletal remains were reburied out of respect leaving only the grave soil (collected simultaneously with the remains) for future study.

In 2015, we initiated a study investigating the soil chemistry and bacterial community diversity (including infectious disease pathogens) of the burial soil samples and their geospatial patterns. We have successfully detected all human-associated bacteria for each burial inhabitant. We have reconstructed the human microbiome for 66 NYABG individuals. Detection of human microbiome profiles gives us insight into individual and ancestral identity, living conditions, and possible causes of death of the corresponding burial inhabitant. Our findings demonstrate the capability to detect human evidence in soil that has been buried for 400-hundred-years. This demonstration serves as proof of concept to explore genomic human aDNA in the NYABG soil samples and other burial soils around the country of similar age (Clinton, 2021). Researchers have acknowledged the human microbiome as our “second genome,” i.e., an additional source of genetic diversity and identity (Grice and Segre, 2012) ***.*** The potential aDNA analysis from the NYABG soil samples will allow us to capture a subset of a historical population (15,000 still buried) and enrich genomic databases with African descended genomic data. By capturing the genomic architecture of this 17th and 18th population, we can perform population genetics analyses to observe evidence of human variation and disease susceptibilities helping us to combat health disparities. We can also contribute this newly generated data to existing databases where people of African descent are underrepresented. We hope to use this investigation as a proxy for the potential to explore other African American burial grounds around the country without disturbing or destroying remains but still learn all that we can about the genetics of African Americans.

### Challenges of studying historic African American remains

Several challenges must be addressed when studying historical remains (skeletal or soil) of African American populations for ethical scientific research with advanced molecular technologies. One challenge is to ensure that research on historic populations is performed in an ethical nature by protecting the sacred ground where they are buried. In addition, legislation must be established to ensure construction or housing development projects do not decimate African American burials (Clinton and Jackson, 2021) ***.*** Often, these projects physically destroy burial sites and erase the existence and contributions of the buried population from history. The lack of burial site protection for African Americans promulgates the idea that they, as a population (alive or dead), are worth less than other populations in America. The lack of protection increases the difficulty for researchers to gain access to these grounds and move forward with investigations. Another challenge of studying historical remains is determining the best research team to engage with the underrepresented community and conduct the research. Performative and helicopter research are two methods of conducting predatory research where the interpretations and generation of data do not benefit the studied population. These types of research promote more harm than good, resulting in the perpetuation of mistrust between marginalized communities and scientific researchers. The appropriate research team for studying African Americans and other underrepresented and marginalized communities are those who perform research for the greater good of the community, serving their needs for increased representation in databases, accurate interpretation of generated data, and ethical applications of the research to better health outcomes. It is paramount to consider the appropriate decision-makers for how to conduct the research. Decisions should be made by educated members of the descendant community, those who are likely closely genetically and culturally related to the studied population. In some cases, the descendant community may not be the local community but genetically related descendants some distance away from the site. A third challenge is establishing where the generated data will be housed and who can access it. We propose that researchers store data in a private repository where access can be controlled by the stakeholders, i.e., the local or descendant population who will directly benefit from the research. Researchers must acknowledge the purpose, potential impacts, and sources for conducting research, generating data and reporting new findings with the scientific community, general public, and community upon which the research is performed (AABA Code of Ethics)**.**

### CASE #2: The Gullah Geechee population of the Carolina Lowcountry

The Gullah Geechee people are an historically important Legacy African American microethnic group residing largely in the Southeastern United States They are a candidate ancestral group to a diverse array of African American peoples across North America. The original migrations of the ancestral Africans moved from staging areas like Charleston, SC and the nearby Sea Island to more inward locales as the United States Frontier was pushed westward. In addition to their geographical isolation (Matory, 2008) ***,*** enhanced retention of African allelic variants and cultural practices, many Gullah Geechee peoples migrated from coastal Carolinas to adjacent regions. Some Gullah Geechee who escaped enslavement, fled to join Black Seminole populations in the Spanish held territory of Florida. Creek Freedman, many derived from Gullah Geechee lineages became refugees on the Trail of Tears to Indian Territory (present day Oklahoma). When the United States government forced First Nations peoples to accept individual land allotments, many Freedmen established predominantly African American towns with other former enslaved African Americans of the Five Tribes. Here they settled together for mutual protection and economic security (Oklahoma Historical Society) ***.*** Black Seminole Freedmen populations also founded communities in northern Mexico, Texas, Oklahoma, and Red Bays Settlement in Andros Island, Bahamas (Holm, 1983) ***,*** escaping Florida after the first Seminole War. The notion of ancestral linkage or the Gullah Connection is commonly affirmed via historical and anthropological records, but little work has been done to confirm this genetically (Paris, 1995; Opala, 2009) ***.*** We have hypothesized that Gullah Geechee genomic and cultural signals proliferate beyond their current geographical territories in diverse African American communities throughout North America (Caldwell and Jackson, 2021) ***.***

The Gullah Geechee homelands of the Lowcountry were the most affluent area of British North America during the colonial period and became an optimal site for African-derived cultures to thrive and adapt. The harsh subtropical climates, malaria transmitting mosquitoes, thick marsh and swamp lands would provide environmental insulation for the amalgamation of Africans to retain and synthesize their own cultural preferences. It also provided an ecological setting to which many Gullah Geechee peoples were preadapted genetically. Intentional admixture was encouraged by Europeans and European Americans to prevent slave revolts among newly arriving enslaved Africans (Scharff et al., 2010)*.* However, many South Carolina slave owners would visit their plantations only as needed to avoid the harsh climates while maintaining the authority needed to ensure profitable production schedules. Physical and social isolation allowed for the unique Gullah Geechee culture to emerge as a synthesis of many African (and non-African) traditions. Their storytelling, veneration of the ancestors, belief in a higher power, and unique cultural attributes were all amplified in the setting of the Sea Islands. The emerging Gullah Geechee peoples, like their creole dialect, represents a unique fusion of Niger-Kordofan and Afro-Asiatic, Indo-European, and Southeast First Nations patterns.

Like their cultural retentions, isolation enhanced the potential for genetic drift in the population. Contemporary lineages from Sapelo Island may reflect the disproportionate influence of a founder, Bilal who became known over time as Bailey among the local Gullah Geechee peoples (Bailey, 1995)*.* As a progenitor Legacy African American population, the Gullah Geechee should have retained ancestral markers with a stronger West and Central African signal compared to other Legacy African American populations whose African signals may have been diluted by more admixture with non-Africans. We suggest that the Gullah- Geechee genomic profiles will show distinct characteristics of endogamy and substructure when compared to other African American microethnic groups as a reflection of their unique history and preeminence. In a recent unrelated study of the Gullah-Geechee (Zimmerman et al., 2021) it was observed that, relative to non-Gullah African Americans from the Southeast United States, the Gullah exhibited higher mean African ancestry, lower European admixture, a similarly small Native American contribution, and increased male-biased European admixture. A slightly tighter bottleneck in the Gullah 13 generations ago suggests a largely shared demographic history with non-Gullah African Americans, as we observed previously (Caldwell and Jackson, 2021) ***.*** Despite a slightly higher relatedness to populations from Sierra Leone (Zimmerman et al., 2021)*,* overall, the studies demonstrate that the Gullah are genetically related to many African populations, representing an amalgamation of West and Central Africans in particular (Caldwell and Jackson, 2021).

A recent study (Zimmerman et al., 2021) confirms that subtle differences in African American population structure exist at finer regional levels. Such observations were reported decades ago (Jackson, 2004; Jackson, 2008)and their validation can help to inform medical genetics research in African Americans and guide the interpretation of genetic data used by African Americans seeking to explore ancestral identities.

Using the Ely-Jackson database, Bert Ely and others (Ely et al., 2006) completed a mtDNA analysis of 78 Legacy African Americans who lived in the Lowcountry and were considered Gullah Geechee descendants. 40% of participant Gullah Geechee had mtDNA migration patterns from West Central Africa, a proportion that resonates with our earlier studies of these peoples (Jackson, 2004; Jackson, 2008)*.* Other Gullah Geechee mtDNA patterns were 23% from Senegambia and 18% from Upper Guinea. Over 30 percent of Ely’s Gullah Geechee participants did not have a mtDNA match with their extensive database of over 4,000 African mtDNA variants (the Ely-Jackson Database), but were clearly of African origin (i.e., most were part of the L megahaplogroup). This illustrates the current limitations of the African-centered reference databases needed for comparative reconstructions of African origins. Although half of the African American participants were able to trace their ancestry to multiple ethnic groups of continental Africa south of the Sahara Desert, Ely and his team (Ely et al., 2006) recognized that autosomal DNA would be needed to determine more information about the probably African ethnic groups of origin because mtDNA was not conclusive enough to determine a single ethnic source of maternal lineage. Ultimately, they suggested that more work should be done to geospatially map African American mtDNA haplotypes. In a more recent mtDNA study of the Gullah Geechee (Fleskes et al., 2021) all had mitochondrial lineages belonging to African haplogroups (L0-L3), with two individuals sharing the same non-African H1cb1a haplotype, while one had a Native American A2 mtDNA.

The geographic isolation of the Gullah Geechee well into the 21st century has allowed them to retain more African ancestry informative alleles and maintain more African cultural retentions than adjacent contemporary Legacy African Americans further inland. Our research among the Gullah Geechee has created a comprehensive analysis of this microethnic group to better understand how they evolved and impacted the broader African American communities. The genomic variance among the Gullah Geechee undoubtedly contribute to the dramatic patterns of health inequities in their region. Remnants of state-sponsored chattel slavery and draconian segregation laws relegated a large proportion of the African Americans to populate the Southeastern part of the United States densely and disproportionately. It is within these settings that various African American microethnic groups emerged and proliferated (Taylor, 2019) ***.*** Generations later, descendants of enslaved Africans are still clustered in the Southeastern states (e.g., the Stroke Belt: North/South Carolina, Georgia, Florida, Arkansas, Louisiana, Mississippi Alabama). In these states, the prevalence of stroke, diabetes, and cardiovascular disease (CVD) are the highest nationwide (Barker et al., 2011; Karp et al., 2016) ***.*** Advances in the control of modifiable biocultural risk factors that served as disease triggers given the genetic backgrounds and comorbidities of the Gullah Geechee (e.g., obesity, hypertension, cigarette smoking, and high salt diets), have produced a decline in stroke related mortality and morbidities. However, data continues to suggest major ethnic disparities in stroke related mortalities among African Americans. Heart disease is the number one killer of African American women (Esenwa et al., 2018) ***.*** Poor CVD health in these communities is exasperated by the history of slavery, social segregation, lack of access to healthcare and healthcare providers, institutionalized racial discrimination, stress, and economic instability. Moreover, institutional levels of inequity, coupled with genomic mediators like the epigenome lead to physiological precursors for stroke (Kramer et al., 2017; Esenwa et al., 2018)*.*

The scientific literature suggests that the high rates of chronic disease in African Americans are caused by the combined and compounded effects of genetic, environmental, and social factors. Yet little is known about the magnitude or geographical distribution of African American genetic diversity, cultural disease catalysts, and population substructure between and within African American populations. Due to their underrepresentation and the present bias toward European and European American genomics, research is needed to understand the effect of multiple genes, epigenetic modifiers, environment, and lifestyle and cultural risk factors that increase susceptibility of these multifactorial disorders (Grundy, 1998) ***.*** We need to be able to apply network analysis and sophisticated computational biology models to depict interactions in African American populations.

In addition to considering the multiple contributing factors that influence chronic disease, our research among the Gullah Geechee suggests that ancestral analysis may uncover evolutionary contributions that have not been considered in other populations because of the ancient, frequently unacknowledged, and often unique genetic underpinnings of populations of recent African descent. Genome‐wide studies (GWAS) have become important genomic tools to use in genetics to associate specific genetic variations with diseases. The method involves scanning the genomes from many different people and looking for genetic markers that can be used to predict the presence of a disease phenotype. Most GWAS are focused on Europeans (52%) and Asians (21%) (Hoffman et al., 2016a)*.* African populations make up less than 1% of the total GWAS studies. The largest African American GWAS study consists of 8,000 individuals while the largest European American GWAS study encompasses 100,000 individuals (Abel and Schroeder, 2020)*.* This means that means that many population-specific pathogenic variants are left undetected. Just as often, many alleles that could provide ameliorative effects for disease phenotypes also remain undiscovered. For example, African Americans are three times more likely to experience kidney failure than European Americans and African American kidney disease tracts clearly with dementia in African Americans (Laster et al., 2018; McAdams-DeMarco et al., 2018)*.* Without the knowledge of the range of genomic diversity in our entire species, and particularly those individuals of recent African ancestry, our efforts to understand human variability adversely affects the control of associated health disparities, exaggerating these disparities over time, limiting the reproducibility of our data, and truncating the significance of our GWAS findings. Including more African-descended populations in genomic research widens the possibilities for more precise clinical application, biomedical treatments, evolutionary insights, and more equitable health policies for every population. Systematically Including African-descended groups takes the scientific community a giant step toward greater parity. For example, recent large studies (Tang et al., 2001) of GWAS for Alzheimer’s Disease in African Americans found eleven novel risk loci, seven of which were rare. Many of the exact genes differed from those identified in European American GWAS investigations. This emphasizes the importance of using genomic studies to assess the higher dementia rates among African Americans and it confirms that the most important genes associated with Alzheimer’s Disease vary between populations even though the deep ancestries of every human population can ultimately be traced to continental Africa.

Finally, our research among the Gullah Geechee suggests that an important avenue for exploring genomic and cultural variation in a geospatially complex and diffuse population such as Legacy African Americans is to study the founding population segments. The Gullah Geechee are an important African American founding population who emerged soon after Africans first were brought to the Carolina Lowcountry (Caldwell and Jackson, 2021) ***.*** Researching such groups can provide important and unexpected insights into disease etiology and inheritance patterns. Two recent studies (Gupta, 2021; Zimmerman et al., 2021)confirms that subtle differences in African American population structure exist at finer regional levels, using the Gullah Geechee as an example, confirming the initial observations of substructure in African Americans in the United States. Such observations can help to inform medical genetics research in African Americans and guide the interpretation of genetic data used by African Americans seeking to explore ancestral identities. The genomics of founder populations can provide explanations for variations seen in complex disease mapping. Such efforts can also track the effects of genetic drift events and historical processes on the population, document regional changes in allele frequencies, identify evidence of cultural adaptations, and monitor the incidence and prevalence of complex disease distributions. Founding events can also be used to locate progenitor populations for contemporary admixed populations. Investigation of founding populations and more inclusive GWAS studies have the potential to capture a wide range of genetic and environmental interaction networks while appropriately contrasting estimates of genetic risk versus environmental or systematic infrastructural risks that perpetuate current disadvantageous outcomes.

**FIGURE 2:**
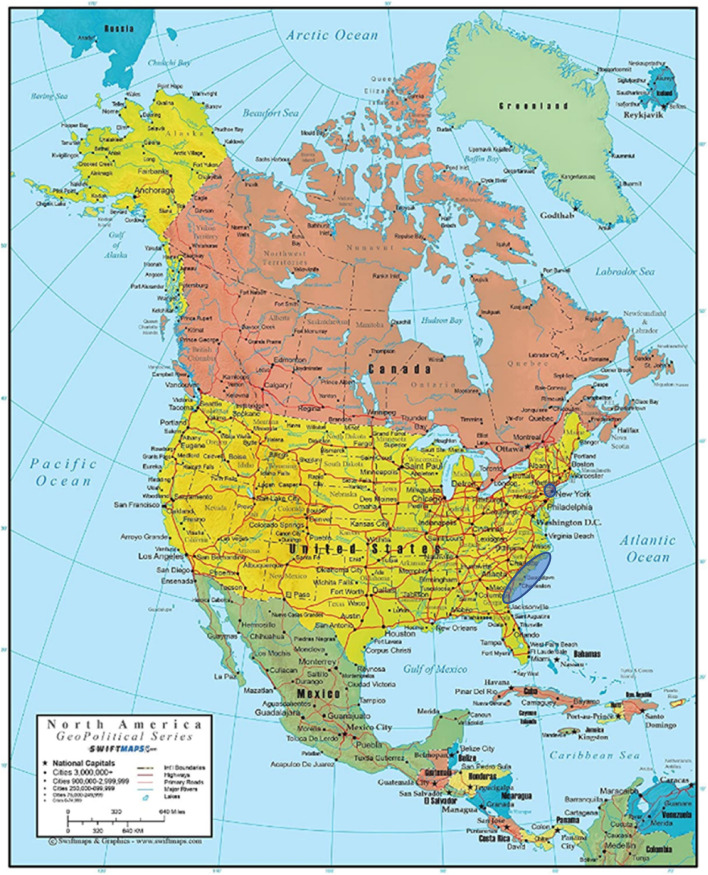
Blue highlighted areas are the geospatial locations of the two case studies reported in this paper. The New York African Burial Ground is located in New York City (Lower Manhattan) while the Gullah Geechee peoples reside along the Carolina Lowlands from Wilmington, NC to Jacksonville, FL.

Figure 2 depicts the geographical ranges of the two case studies presented in this paper.

## Core issues emerging from the case studies

Recently, the American Anthropological Association admitted to the racist attitudes and perceptions permeating the discipline with respect to the indigenous peoples of the Americas. As Gupta (Jackson, 1998) writes “*Since its inception, the history of American anthropology has been intertwined with a record of extractive research conducted on the Indigenous communities. Anthropologists have often assigned themselves the status of ‘expert’ over the cultural narratives and social histories of the first cultures of the Americas. As ‘experts’ many anthropologists have neither respected the endogenous knowledge systems and community contributions of Native Americans (or other indigenous peoples) nor addressed the intended and unintended impacts of anthropological research on those communities. Some anthropologists now acknowledge the harms that have been caused by researchers in the discipline, but it remains the case that anthropology must explicitly address the need to change its ways*.”

The same should be said for the treatment of Legacy African Americans. African-descended peoples on the continent of Africa and throughout the African Diasporas have also been historically maligned and neglected by the scientific community. Even in contemporary genomic studies it is rare to hear an emic perspective of the African American genomics interpretation. By emic, we are referring to its anthropological use in denoting an approach to the study or description of a particular language or culture in terms of its internal elements and their functioning rather than in terms of any existing external framework. For our purposes in this manuscript, each of the authors is a member of the African American community with extensive research in community engagement, historical narratives, biological anthropology, and genomics. Our perspectives are indigenous, internal to the culture, and emic capturing the sensitives and diversities within our population.

The importance of African-descended populations in genomic studies and the development of a truly global genomic database cannot be underestimated. Given the evolutionary origins of humanity in Africa, we have long argued that the various state-sponsored human genome projects should have long ago focused on the genetics of recent African descendants to adequately reflect a more plausible template for our species (Kararach et al., 2011)*.* A quarter of a century later, we still lack an adequate African-centered database for our species. Genomic research in Africa has a long way to go and genomic research among African Americans should be more advanced than it is presently, given the physical proximity and accessibility of this segment of American society. Researchers working in Africa have only studied between 5,000 and 10,000 whole genomes from the continent, compared with as many as 1 million whole genomes worldwide. Africa has received less than 1% of the global investment in genomics research and clinical studies. Genomics studies in Africa could contribute significantly to research worldwide in understanding our species since all our lineages ultimately trace back to Africa where *Homo sapiens* emerged some 300,000 years ago. Even those human lineages who left continental Africa over the past 80,000 years ago and spread across the planet carry only a subset of human genomic diversity. As a result of this evolutionary history, the people of Africa today carry more genetic diversity than those of any other continent. There are segments of human genome that can only be studied in Africans since these are the only populations within in which these unique sequences and genomic components are found.

Furthermore, populations of recent African descent are a growing segment of the world community, and these populations tend to be younger, so hopefully, African-descended individuals and communities will be around longer to benefit from todays and tomorrow’s genomic innovations. In 1950 the population of Africa was 177 million and it grew 7.6 times to more than 1.341 billion in 2020. Africa is the continent with the youngest population worldwide. As of 2021, around 40 percent of the population is aged 15 years and younger, compared to a global average of 26 percent (Micheletti et al., 2020)*.* Africa is quickly recovering from the destructive population losses associated with centuries of extractive enslavements facilitated by wars, exploitative colonialization by European and Arab powers, and years of local political mismanagement precipitated by low educational levels.

Legacy African Americans are not a genomic substitute for continental Africans as much autochthonic continental African genomic variation was lost among African Americans during the genetic bottlenecks of the transatlantic Middle Passage, the subsequent ravages of American Slavery, and the generations of forced gene flow with non-Africans. Instead, the justification for studying the genomics of Legacy African Americans stands independent and yet is connected to the need for comprehensive studies of African genetic diversity. In African Americans we have the unique opportunity as researchers to study the effects of well-specified gene-environment interactions on a historically socially restricted population that represents an amalgamation of West, West Central, and Southeast African peoples with modest gene flow from select non-African groups, primarily North Atlantic and Iberian Europeans and eastern Native American peoples.

The mobility of these early enslaved Africans was extremely circumscribed, largely following the forced migrations to North America. Countering this lack of geospatial movement was the fact that enslaved Africans represented, from the start, a broad array of geographically and culturally distinct African peoples. Initially these diverse Africans sorted themselves by their original African ethnic groups or their closest affiliates on the African continent. The initial retention of original identity provided a template for resistance among the survivors and their immediate descendants (e.g., the nearly constant slave rebellions and uprisings were often organized and implemented along African ethnic affiliation) and sexual selection (e.g., especially female-based mate selection may have been based on African ethnicity and religious preference). The effectiveness of this self-sorting was density dependent. Initially we observe genomic aggregates based upon original ethnicity, but these aggregates were strongly discouraged by slave honors because of the enhanced potential for rebellion mentioned previously. Where there were larger numbers of enslaved Africans, such as the big plantations of the Southeastern and Mid-Atlantic regions of North America and in the urban areas of the American colonies such as New Amsterdam/New York City, African genomic integrity and cultural preferences could be retained longer and more cohesively. Over time, however, within the context of institutionalized, multi-generational enslavement, self-identities were transformed, and the original African ethnic affiliations gave way to new localized identities. This is the genesis of the many microethnic communities of African Americans that today can be found throughout the homeland and satellite territories of the African-descended peoples of the Americas. African Americans follow this same generalized population biology pattern of initial fusion followed by transformation and subsequent fission.

Of the limited number of comprehensive genomic studies done on African Americans, we can already see the promise of genomics to reveal major insights. A major DNA study (Loshin, 2002) recently shed new light on the fates of the more than 12.5 million Africans who were enslaved and traded to the Americas between 1,515 and the mid-19th century. More than 50,000 people took part in the study, which was able to identify more details of the “genetic impact” the trade has had on present-day populations in the Americas. The study laid bare the consequences of rape, maltreatment, disease, and racism. More than 2 million of the enslaved men, women, and children died enroute to the Americas. But the interpretation of the results in this major paper were ahistorical and overemphasized the presumed genetic affinities of African Americans to modern day Nigeria (Jackson, 2021)*.*

Despite the errors, if African Americans genomic studies can be a rich source of insight into human evolutionary biology and evolutionary medicine, who should own the resulting data? The question of ownership of genomic data is fraught with cultural nuance and interpretation. Data ownership refers to both the possession of and responsibility for information as ownership implies power as well as control (Githaiga, 2021) ***.***

For African-descended populations, there is no single cultural mandate among the indigenous peoples of Africa. For example, on the question of land ownership, indeed, the East African Community (Kenya, Tanzania, Rwanda, Burundi, and Uganda) is currently struggling with contentious traditional cultural perceptions of land that have defined land ownership, use and access (Stokstad, 2019) ***.*** Genomic variation is a valuable resource. So, perhaps it is more analogous to trees. The oldest dictums from a collective of East African ethnic groups suggests that whoever plants a tree, owns that tree and the products of that tree (e.g., the fruit, the oil, the sap, the lumber). Even with changes in land ownership, the tree belongs to whomever planted it and his or her descendants. This is an appropriate metaphor for the control of genomic data generated in the process of biomedical research and ancient DNA studies. The data clearly stay with the population of origin and their descendants; they own the products of their ancestral trees. Descendant communities must also be engaged in the analysis and interpretation of these data. While their ownership does not preclude non-indigenous access to data, the lines of responsibility must be grounded in the African American community. The past bio-colonial paradigm of external ownership of African American genomic resources should be rejected.

Within traditional Africa, communities are generally structured hierarchically such that their organizational structure serves somewhat as a buffer against genetic exploitation. And yet, African genomic studies here have too frequently been characterized by ethical dumping, in-and-out helicopter science, and over extrapolation of limited data by Western scientists with few ties to the local communities. Researchers gathered samples with scant regard for informed consent and without giving back information and other resources to the communities they studied. Outside of structured communities in Africa, the threat of genetic exploitation was expected to be protected against by local governments. These protections have clearly not been fully effective, however. A recent prominent example has been the United Kingdom.‘s Wellcome Sanger Institute. Here, whistleblowers in 2020 privately accused Sanger of commercializing a gene chip without proper legal agreements with partner institutions and adequate informed consent of the hundreds of African people whose donated DNA was used to develop the chip (H3Africa, 2021) ***.*** The institute confirmed that it did not commercialize the chips or profit from them but admitted that its relationship with some African partners has been “disrupted.” Stellenbosch University in South Africa has demanded that Sanger return these samples. Sanger’s mishandling of this extensive genomic sampling effort will likely contribute to the ongoing erosion of trust between researchers and diverse African people, setting back genomic research that could have been of benefit to Africans and their recent descendants. This controversy with a major genome research center will inevitably retard the study of African genomics because it will amplify the existing distrust between African communities and the Western scientific establishment. However, Africans have begun to initiate their own studies, aided and inspired substantially through the Human Heredity and Health in Africa (H3Africa) Initative (Jegede, 2009) led by Charles Nohuoma Rotimi who is the Director of the Trans-National Institutes of Health (NIH) center for research in genomics and global health. It is these initiatives among both Africans and African Americans that will provide the best protection against a continuation of past genomic abuses (Thompson et al., 2003; Pellegrino et al., 2007) ***.***

Additionally, the development and expansion of scientific expertise among Africans and African Americans in the genomic sciences will allow the development of significant capacity building within these segments of the scientific community and the development of trust with the larger social and cultural communities from which these new scientists have emerged. True informed consent can only come from a foundation of trust based on correct understanding. Trust is built on shared experiences, shared expectations, the anticipation of predictable outcomes, and is a central part of all human relationships. Informed consent emanates from an educated understanding of the issues at hand, an awareness of the limitations of the technology in use, an appreciation of the meaning of the results generated by the research, and past evidence of mutual goodwill among the researchers and the researched. The specifics of informed consent will vary across the range of a species, indeed across the range of a stratified subset of the species. Among African Americans, informed consent may vary across North America since the perceptions of key cultural components also diverge regionally. For example, the recognition of the rights of the dead and the veneration of ancestors vary across the geospatial range of African Americans. In African American cultures with strong African retentions, such as the Gullah Geechee peoples, the veneration of ancestors is strong and while augmented by a belief in a supreme being, prayers and/or sacrifices are also offered to the ancestors who may be conceived as minor deities. In these communities, the disposition of skeletal and dental remains, tissue samples, and DNA samples may take on additional significance. Only through careful ethnographic inquiry and structured survey methods (e.g., the collection of qualitative and quantitative data from the actual African American communities of relevance) can we begin to document the nuance of diverse perspectives evident among African Americans with respect to genomic studies. In spite of the regional substructure among African Americans, there does exist a *“collective cast of mind.”* (***Cited in*** (Wolinetz and Collins, 2020)) on the many issues that determine what is collectively valued, who the people consider themselves to be, what priorities define them as to who they are, and how them perceive themselves in the larger society. Without these data providing an authentic and collective voice of the people, researchers are not only sampling blindly and magnifying disparities, but they are denying African Americans the autonomy as laid out in western ethical principles (*see* (Sanders, 2021))*.*

The troubling victimization and exploitative history of Legacy African Americans by the early biomedical and genomic science studies of the United States lays a challenging foundation for ethical future studies. Researchers must be even more careful in acquiring and documenting fully informed consent from African American individuals and communities and providing any requested feedback on the research results and needed educational opportunities. As the African American community collectively becomes more astute as to the nature of scientific research, additional ethical requirements will emerge, particularly for genomic studies. For example, the technological innovation of CRISPR Cas9 (clustered regularly interspaced short palindromic repeats and CRISPR-associated protein 9 now permits genome editing (also known as gene editing) giving scientists the ability to directly manipulate an organism’s DNA. In 2014, one of the first cases of applying this technology to humans was the editing of the genome an African American with sickle cell anemia (Frangoul et al., 2021) ***.*** This disease afflicts millions of people around the world, most of them of African descent. Some 100,000 African Americans are afflicted with the disease. After 6 years of work, that experimental treatment was approved for clinical trials by the United States Food and Drug Administration, enabling the first tests in humans of a CRISPR-based therapy to directly correct the mutation in the beta-globin gene responsible for sickle cell disease (Graves et al., 2022) ***.*** Yet, the application of CRISPR cas9 also reduces population variation, which, according to evolutionary theory, increases a populations vulnerability to extinction. As CRISPR-based interventions become more widespread and of public health significance, the ethical and evolutionary implications of diminished population genomic variability in the quest for immediate improvements in individual health will have to be reconciled. Undoubtedly, African American communities will figure prominently in these discussions because of the historical legacy of western science seeking pathology (in the context of disease alleles) in Black bodies.

Clearly, the larger scientific community has an obligation to promote researchers from underrepresented communities at all levels of genomic sciences. This is, in fact, the best response to past wrongs, and the strongest deterrent against future ethical abuses. Recently, Graves and others (Graves and Goodman, 2021) ***.*** called for a new agenda to address inequality in science. In this call, they stressed the need to attract individuals who have been historically excluded from participation in science and highlighted the importance of engaging in substantial work to overcome the longstanding obstacles to their full participation. This call cannot be overemphasized: multidimensional African American involvement in the genomic sciences is essential to make up for the current deficiencies in the global database and, just as importantly, to rectify the inadequacies in a comprehensive understanding of the genomic ramifications the African American experience in North America. Accurate, historical and culturally-contexed interpretations of the genomic data are as important as the raw genomic data themselves. In fact, to have the latter without the former provides little good for the African American population. In the authors experiences at Howard University, we have witnessed the value of interdisciplinary input in genomic science interpretation. We also have had the firsthand opportunity to work over a number of years with the two case studies presented below, the New York African Burial Ground and the Gullah Geechee peoples of the Carolina Lowcountry, evaluating both from emic perspectives.

## Origins of African American mistrust in medicine and its consequences for genomic studies

North American patterns of institutionalized racism, state sponsored segregation, and social disenfranchisement in genomics are reflected in the historical medical practices of the country. Thus, the patterns of inequality remain a tenacious part of contemporary research practices and perceptions. Concepts such as race, ancestry, genetics, access, equity, equality, and medicine are intertwined and intractably interconnected due to the pervasive historical pattern of exploiting race as a biological construct (***see*** (Washington, 2006)) ***.*** In *Medical Apartheid*, Washington (Thompson et al., 2003) describes the dehumanizing processing of enslaved Africans and their African American descendants upon their arrival in the Americas resulting from the transatlantic and domestic trades as they were sold to new “owners”. Inadequate personal privacy, lack of sanitation, overcrowding, stark nutritional deprivations, and other detrimental public health conditions for enslaved Africans and their African American descendants meant enhanced exposures to infectious diseases from Europe, the Americas, and Africa, compounded by the disorders of nutritional deficiencies, the psychological and physical traumas of enslavement, and the enslaved persons preemptive status as experimental models for early biomedical studies. This was done without the documented consent whatsoever of participants and these studies were enacted without the researchers understanding for or appreciation of the ancestral backgrounds or population substructure of African Americans. Black bodies were poked and prodded, surgeries were performed without available anesthesia, and known therapeutic medications were withheld. Simultaneously, non-traditional and herbal based medicinal practices were banned in Legacy African American communities. Stories of the “strength” and “lack of pain” experienced by African American women in childbirth plague their level of care in Labor and Delivery wards today. If enslaved African Americans complained about their aliments, these nascent physician-scientists responded according to the directions of the plantation owner whose goals were consistently to maximize their economic profits. This resulted in veterinarians “practicing” on humans and harmful “quick-fixes” done more often than necessary. Early experimental studies on exploited, enslaved, and newly freed African Americans were used to bolster tainted theories about European and European American supremacy in intellect and humanity and have set the historical template for the ethical challenges we currently face in studying the genetics of these continually marginalized communities. The prejudices and beliefs of this historical time has prevailing implications, even unconsciously in contemporary western medical spaces.

The mechanisms that have contributed to the marginalization of Legacy African Americans and their descendants, the importance of performing ethically responsible research on underrepresented populations, and the consequences of performing more inclusive, unbiased research on historic and contemporary African Americans emerge directly from the case studies we present.

Learning more about the genetics of historic populations, particularly, those buried in the NYABG helps us better understand the genetic identities of free and enslaved Africans, genetic adaptation due to the world’s most extensive forced migration pressures, and genetic diseases that affected a historical population. In addition, increased knowledge of historic African American genomics allows researchers to comprehend better the genomics of living African Americans. Illuminating the genomics of African Americans is essential for several reasons such as: 1) it provides a multi-dimensional sense of identity, genomic and ancestral, that was severed by the Transatlantic Slave Trade, 2) it reveals the diversity within continental Africans, ultimately contributing to a greater understanding of all humankind and 3) it contributes to the paucity of African descended peoples in genomic databases to be used by medical professionals to make more informed diagnoses and treatment plans as we move into the age of precision medicine.

A premier concern in exploring these insights is ensuring ideal conditions (financial, ethical, and legal) are met to study African American genomic research appropriately. First, funding agencies must see the value in studying African and African American populations with an inclusive benefit for them and their descendants. Ethically responsible research to respectfully study underrepresented groups must become standard practice. Finally, legally, protections must be set to ensure the safeguarding of African American biological samples, remains, and genetic data (Jackson et al., 2021) ***.***

The need to protect African bodies was proven necessary upon the arrival of the first enslaved Africans to the United States based on the understanding that chattel slavery was dehumanizing and immoral. The need to protect African bodies from illegal biological research was a simultaneous necessity as many were purchased for the sole purpose of medical experimentation to advance the reputation and career of the purchasers (Thompson et al., 2003)**.** An early example of using African bodies against their will and exploiting biological processes for financial and economic gain is in the work of J. Marion Sims, the “Father of Modern Gynecology” during the mid-18th century. He was praised in the medical world for his advancements in vesicovaginal fistula treatment and the first gallbladder surgery, which he developed and practiced on enslaved African women. However, it was not until recently that years of controversy stemming from Sims’ ethical practices around discovering these advancements through his unorthodox experimentation on enslaved women led to a change. While there was no compensation for African Americans, retribution came in 2018 when New York City finally removed his statue from Central Park across from the New York Academy of Medicine (Walloo, 2018) ***.*** Another example involves Georgia physician W. H. Robert and his inclination to amputate the limbs of enslaved Africans for minor injuries as demonstrations for medical students. He believed that students should “hesitate much less to remove a limb … , if he be slave, than if he be a free man, and especially a white man.” This advice was based on Robert’s observation that the surgical pain felt by an enslaved person was negligible, minor compared to what a white man facing the procedure would feel (Thompson et al., 2003) ***.*** The idea that people of African descent do not possess the capacity to feel pain at the same intensity as white people still resonates throughout the medical industry today. Studies found that when a Black person enters an emergency room with pain like a broken bone and then a white person enters an emergency room with the same ailment, the Black person will receive a lesser dosage and even sometimes an inferior treatment. A 2016 survey of 222 white medical students and residents revealed racial bias in pain perception and accuracy of treatment, including less effective pain-relieving options (Hoffman et al., 2016b) for African Americans. Notions such as this are the basis for large-scale socio-economic crises, like the opioid epidemic.

### Scientific research on African American remains

As identified in the studies of the New York African Burial Ground, just as enslaved Africans were controlled during their lives, European enslavers and public officials carried over this control even after their death. The need to protect African remains became necessary the moment they were buried. Misusing African remains has been demonstrated across medical colleges in the United States during the late 1700s and 1800s (Shultz, 2005; Royes, 2020) ***.*** Employees of medical colleges, medical students, and instructors would illegally dig up the cadavers of African Americans for anatomy instruction. The remains were used without the knowledge or permission of the person or their living relatives. The bones were never replaced after their teaching purpose was fulfilled. The affected families were never compensated. Furthermore, the bones of the unearthed individuals were never acknowledged for their contribution to scientific advancement (Thompson et al., 2003) ***.***

The Medical College of Georgia’s (MCG) participation in “grave robbing” is of relevance. In 1989, a construction project renovating the Old Medical School building uncovered an estimated 9,000 human bones (350–450 people) buried in the basement. Most of the remains were taken from a predominantly African American cemetery, Cedar Grove, years before dissection of bodies became legal in 1887 (Taylor, 2019)**.** However, even in this blatantly illegal and morally corrupt act of stealing bodies, the MCG did little more than recognize their predatory past. Only by revisiting the MCG discovery (along with other exploitative investigations of Black bodies and mishandling of their remains) and noting where more appropriate, respectful, and ethically responsible actions could have been taken can we truly understand the unfortunate foundations of the United States medical industry.

The challenge of studying historically underrepresented populations, particularly African Americans, is that their existence (in life and death) has been undervalued. As we have seen throughout history, if a group is undervalued, there is less investment for scientific researchers and physicians to benefit that group. Benefits include but are not limited to using informed consent (by researchers and medical professionals), allowing individuals to make autonomous decisions about their medical procedures, receiving medical care using the same methods that have been developed with the reluctant participation of enslaved Africans, and assured protection for burials from graverobbers or overzealous medical students. Unfortunately, the limited investment in African American research results in a failure to learn all we can about the genomic makeup of an underrepresented group in scientific and medical research. Further, because the limitation stunts our understanding of the genomic variation and diversity in African descended peoples, the population whose origin is located on the same continent as the inception of the*Homo*species, we fail to learn all that we can about the entire human population.

### Absence of African genomic data in global databases

There are exceptions to the undervalued condition where historically marginalized groups, in this case, African Americans, are commoditized for their biological genomic data. Usually, these exceptions occur when research is performed to benefit European researchers and patients. An example of this exception is seen in the increasing thirst of commercial DNA testing companies to enrich their databases (Jaiswal and Halkitis, 2019)**.** The origins of American medicine and the direction of medical practice are driving factors for inequities in our healthcare system and scientific research. As researchers work to expose, address, and dismantle how deeply entrenched biases have shaped scientific research and medicine, we are forced to consider how we presently deal with race, access, and health disparities. The reluctance of many African Americans to engage with the American medical system stems from a generational pattern of historical mistrust of the system and its founders (Gamble, 1993; Suite et al., 2007; Sirugo et al., 2019) ***.*** We are approaching a fork in the road, where if researchers continue down the current path, where African descended people make up roughly 2% of global genomic database contributions (Popejoy and Fullerton, 2016) ***,*** we will reach a point where African Americans are exponentially lagging (even more than the present status) in genomic research regarding health outcomes and the potential for personalized medicine applications. The large gap between the number of European participants in genomic databases and all other groups results from historical, cultural, scientific, and logistical factors sustaining bias in genomic research (Atutornu et al., 2022) ***.*** Genome-wide association studies (GWAS) surveys show that over 70% of samples come from the United States, Iceland, and the United Kingdom. Choosing the path less followed means embarking upon a new Frontier where geographically and ethnically diverse genomic databases serve as an enriched reservoir for more accurate and less biased scientific research. Human genomic diversity between African genomes and the rest of the world results in differences between the variants associated with specific disorders and genes, making it more challenging to find the link between genetic variants and disease in African descended peoples. This challenge means that causal links between variants and disease cannot be trusted in medicine if the data upon which the diagnosis is formulated does not include populations from diverse ancestral backgrounds (Coles and Mensah, 2017)**.** If an adjustment to this new path is not made, African Americans will continue to exponentially lag other groups in the race to precision medicine, or worse, be given the wrong genetic diagnosis or risk profile for disease. They will continue to be disadvantaged in genomic research opportunities leading to better overall health and access to personalized medicine applications, gene therapies, and pharmacogenomic benefits (Atutornu et al., 2022) ***.***

The historical mistrust between the African American community and the healthcare industry is a crucial factor contributing to missing data in genomic databases (Coles and Mensah, 2017) ***.*** Tackling this predicament requires the continued rebuilding of confidence at every level of healthcare to demonstrate its investment in the lives of African Americans. While an exact solution is unclear, we hypothesize that once developed, it will take years of application to rebuild trust among African Americans. Researchers are working to combat the paucity of diverse data among living African Americans in genomic databases through initiatives such as the H3Africa consortium (Bentley et al., 2020) and the *All of Us* research program (Department of Health and Human Services, 2019)*.* Others are working on grasping a more robust understanding of African American genetics through studying African American remains. One way to combat the missing data issue is by analyzing historic African American genomes. With the permission of the descendants of these buried populations, researchers can address the dire need to enrich genomic databases in two ways. The first is to increase the numbers of African descended genomes in the databases, and the second is by widening the breadth of information that can be learned about a population by studying individuals who lived hundreds of years ago. The relatedness of individuals in a population coalesces as you travel backward in time and thus gives researchers a broader scope of the genetics of living descendants without needing their samples directly. Genomic data from historic remains gives us insight into the health disparities, genetic variation, and disease susceptibilities of living Legacy African Americans. Research on historical remains provides a window into the genetics of living African Americans circumventing this historical mistrust and fear to ensure a future for access to precision medicine for this underrepresented group. Pushing human remains research forward, we at Howard University set out to observe human evidence in NYABG burial soil samples that have been buried for four hundred-years.

### Ethical influences on genomic testing of African Americans

Prior to the inclusion of ethical principles in the routine training of physicians and scientists, enslaved and newly freed African Americans were disproportionately represented in unregulated experimental studies and were the targets of eugenic hypotheses ***.*** Once application of the ethical principles of autonomy, informed consent, privacy/confidentiality, beneficence, nonmaleficence, and justice became commonplace in western science, the collection of global genomic databases became overwhelmingly comprised of the genomic data of peoples of North Atlantic European ancestry. This current fact presents continuing limitations for all other (non-European) peoples and the extent of their deficit is proportional to their degree of difference from this North Atlantic European standard. The impact of the underrepresentation is particularly acute for associated health implications, for inadequate genomic and medical research lays a foundation for the perpetuation and amplification of current health disparities among the most disenfranchised. Populations of recent African descent, for example, have greater genetic variation when compared with other non-African populations. African Americans are an accessible population for capturing a proportion of the genomic diversity of Africa. The failure to include African Americans in genomic studies may lead to increased health disparities (Jackson, 1997) ***;*** what is not known cannot be properly addressed, and vital ancestral history will continue to be missed in these communities. While race is not a genetically meaningful category, its social ramifications continue to impact biology through the enactment of racist policies and practices which result in inequities in areas such as healthcare. As we learn more about the fine mapping and interactions of ancestral origins and their correlated disease risks, researchers will be restricted in their capacity to address health disparities, evaluate appropriate applications for precision medicine, and understand the broad landscape of the human genome with such a limited and skewed global genomic database. While these limitations were recognized 25 years ago (Jackson, 1997; Jackson, 1998) ***,*** the genomic community has been slow to address this equity issue.

## Conclusion

Given these core issues, how do we forge a research agenda that addresses the expanding marginality of underrepresented groups such as Legacy African Americans (and African-descended peoples in general) (Rogers and Lange, 2013) in the face of rapid technological advances in genomics and the increasingly direct applications to genomics to clinical diagnostics and therapeutic intervention (e.g., CRISPR Cas9 gene therapy)? We posit that there is important urgency to address the current paucity of Legacy African American genomics specifically and African genomics in general. It is necessary to expand the scope and volume of inclusion for non-European populations to ensure equity in healthcare. Today, all humans alive on Earth share a common ancestor who can be traced back to continental Africa (Cann et al., 1987) ***.*** Human residence has been the longest in Africa and the original population sizes were larger than elsewhere. Additionally, Africa alone comprises at least 11 ancestral groups compared to 12 ancestral groups in the rest of the world (Kwok, 2009) ***.*** With the deepest evolutionary history and the greatest diversity, African genomes can tell us more about the health and existence of humankind than any other population. Genomic databases must be enriched with African descended genomes to paint the most accurate picture of who we are as a species. Perhaps efforts should be made to refine the content of current genomic databases to represent the entire human population accurately. As a step toward parity in genomics, what if databases were 90% African and 10% all other populations? This formulation would be a means to recalibrate our assessments to make them evolutionarily more profound and reflective of a broader cross-section of our species diversity. Such enhanced representativeness is also needed for the future endeavors of our species, particularly genomic modifications that will be needed to make human life on other planets sustainable. We may already have among our species the allelic variants and epigenetic markers that could augment our future extraterrestrial existence.

The most insidious shortcoming of missing genomic data from non-European populations however is the harm it poses to the health and survival of non-European peoples. Due to the paucity of genomic data on African American populations, reportedly “rare variants” do not accurately reflect the overall data but are a product of the bias due to a lack of diversity in genomic research. The absence of data leads to misdiagnosis of the origin of the disease or disorder. Additionally, if non-European populations are not adequately represented in genomic research, they cannot access its benefits, such as gene therapy and precision medicine, including pharmacogenomics that they contribute to as taxpayers. All in all, adequate representation in genomic databases translates to better and more equitable health outcomes and preventative treatment for all people. The rationale for inclusion is clear and the mechanisms needed to ensure that this inclusion is ethical are feasible. What we now lack is the will to implement these important innovations.

## Data Availability

22 publicly available databases were evaluated to create the metadatabase used in this study. Links to these individual databases are provided in the text. Further inquiries about the metadatabase can be directed to the corresponding author.
